# Point-of-sale marketing of heated tobacco products in Israel: cause for concern

**DOI:** 10.1186/s13584-019-0316-6

**Published:** 2019-05-27

**Authors:** Bonnie Halpern-Felsher

**Affiliations:** Division of Adolescent Medicine, Department of Pediatrics, Stanford University, Palo Alto, California, 94304 USA

**Keywords:** Tobacco, Marketing, Adolescence, IQOS, Electronic cigarettes, Regulation

## Abstract

Understanding how PMI markets IQOS at the point-of-sale in Israel is critical to determining whether marketing practices adhere to regulations and appeal to groups most at risk at initiating new tobacco products, such as adolescents. An article by Bar-Zev, Levine, Rubinstein, Khateb, and Berg (2019) examined the marketing of IQOS in retail stores in Israel. They found that while no free samples or promotions were provided at the point-of-sale, IQOS and their related HEETS (HeatSticks) were placed near youth-oriented merchandise and in prominent locations easily seen by youth. Further, package colors were used to indicate tobacco flavorings and strength, and retailers described the IQOS products as being less harmful, a cessation device, and not producing smoke. These findings are concerning given numerous studies linking marketing of novel tobacco products, product misperceptions, and subsequent tobacco use. Studies are needed to ensure that the marketing of IQOS, including the use of package colors, product placement at point-of-sales, and other product characteristics are significantly reducing harm and risk of tobacco-related disease to IQOS users, and that the health of the population as a whole, including those not using IQOS or other tobacco products, will not be harmed. Until such evidence is available, caution is warranted and regulations needed in the marketing of these novel products.

Novel tobacco products continue to proliferate and infiltrate the market, varying in nicotine content, heating mechanisms, devices, and flavors, and pushing the bounds of tobacco regulation [1]. One such novel tobacco product is the heated tobacco product (HTP), also known as heat-not-burn. While HTPs have been in development since the 1960s, their widespread marketing and distribution occurred more recently. These products heat rather than burn the tobacco, thus supposedly resulting in no ash, fire, smoke or combustion. HTPs are often marketed as safer or reduced risk tobacco products, despite no independent scientific evidence that they are less harmful, nor studies ensuring that marketing claims of reduced harm or reduced risk will not ultimately increase use and have a negative effect on public health [2, 3].

Phillip Morris International’s (PMI) HTP, the IQOS, contains a separate heated tobacco unit or heat stick, also referred to as HEETS, a holder and a charger. IQOS come in a number of flavors, often signified by package colors. The IQOS has been on the market in over 30 countries, including Korea, Japan, Italy, Germany, and Switzerland, and PMI just received authorization to sell IQOS in the United States. In December 2016, IQOS came on the market in Israel.

Israel regulates IQOS as a tobacco product, restricting its ads on billboards, TV and radio; and requiring signage at point-of-sale indicating the minimum age of purchase, which is age 18 in Israel. However, there are no advertising or marketing restrictions at the point-of-sale. Understanding how PMI markets IQOS at the point-of-sale in Israel is critical to determining whether marketing practices adhere to regulations and appeal to groups most at risk at initiating new tobacco products, such as adolescents.

Bar-Zev, Levine, Rubinstein, Khateb, and Berg (2019) [4] published a pilot study, “IQOS point-of-sale marketing strategies in Israel: A pilot study,” whereby they adapted an existing surveillance tool for assessing five dimensions of marketing of IQOS in 15 IQOS retail stores: store characteristics, products sold, price, promotional strategies, and product placement. Bar-Zev and colleagues found that no free samples or promotions were provided at the point-of-sale, and that IQOS were twice as expensive as other tobacco products. These findings are important to note, as there is clear evidence that people and in particular youth are more likely to use a tobacco product when exposed to promotional materials [5–10].

However, there were several concerning findings from the study published by Bar-Zev and colleagues. The study found that IQOS products and their related HEETS were placed near youth-oriented merchandise, and in prominent locations easily seen by youth. Further, package colors were used to indicate tobacco flavorings and strength, and retailers described the IQOS products as being less harmful, a cessation device, and not producing smoke. There is great concern that such marketing will translate to lower perceptions of harm and increased appeal among not only tobacco users, but non-users, and could result in nicotine-naïve youth initiating tobacco use with IQOS [2, 3]. We have seen this pattern for other novel tobacco products, such as electronic cigarettes. Numerous studies have shown that adults and especially youth misperceive newer tobacco products when they come to market [11, 12]. For example, youth believe using electronic cigarettes will confer significantly less harm with respect to disease and death, but also addiction, compared to cigarettes and other combustible products [13, 14]. Further, youth, including those who have never used any tobacco, indicate being more likely to use electronic cigarettes [13, 14]. Youth exposed to ads for novel tobacco products report the products appeal to them, and such beliefs translate into increased likelihood of use [15]. There is also scientific evidence showing that both adults and youth associate colors with specific characteristics, and that tobacco packaging colors influence consumer perceptions of the tobacco product’s taste and health risks [15–17].

## Conclusions

The findings from Bar-Zev and colleagues [4] as well as the related literature on marketing of other novel tobacco products suggest that the IQOS tobacco product can ultimately lead to increased use especially among youth. Currently there are few studies on the short- and long-term health effects of IQOS alone or compared to cigarettes or other tobacco products. Nevertheless, even if studies do pan out suggesting that IQOS confer less harm, the overall risk of youth initiation of tobacco through IQOS is of concern. Studies are needed to ensure that the marketing of IQOS, including the use of package colors, product placement at point-of-sales, and other product characteristics are significantly reducing harm and risk of tobacco-related disease to IQOS users, and that the health of the population as a whole, including those not using IQOS or other tobacco products, will not be harmed [18, 19]. Until such evidence is available, caution is warranted and regulations needed in the marketing of these novel products.

## Data Availability

N/A

## Abbreviations

FDA: Food and Drug Administration
HEETS: (HeatSticks)
HTP: Heated Tobacco Products
PMI: Phillip Morris International
US: United States
